# Supplementary material for: Real-Time Single-Molecule Studies of RNA Polymerase–Promoter Open Complex Formation Reveal Substantial Heterogeneity Along the Promoter-Opening Pathway
Source: J Mol Biol. 2022 Jan 30;434(2):167383. doi: 10.1016/j.jmb.2021.167383 (PMC8783055; doi:10.1016/j.jmb.2021.167383)
Supplement: Supplementary data 1 [file mmc1.pdf]

Supplementary Information

Real-time single-molecule studies of RNA polymerase–promoter open complex formation reveal substantial heterogeneity along the promoter-opening pathway

Anssi M. Malinen[a,b], Jacob Bakermans[b], Emil Aalto-Setälä[a], Martin Blessing[b,c], David L.V. Bauer[b,d], Olena Parilova[a], Georgiy A. Belogurov[a], David Dulin[b,e,f], Achillefs N. Kapanidis[b,g]

Correspondence: Anssi M. Malinen (email: anssi.malinen@utu.fi) or Achillefs N. Kapanidis (email: achillefs.kapanidis@physics.ox.ac.uk)

[a]Department of Life Technologies, University of Turku, 20014 Turku, Finland
[b]Biological Physics Research Group, Clarendon Laboratory, Department of Physics, University of Oxford
[c]Max Planck Institute for the Science of Light, Staudtstraße 2, 91058 Erlangen, Germany
[d]RNA Virus Replication Laboratory, The Francis Crick Institute, 1 Midland Road, London NW1 1AT, UK
[e]Junior Research Group 2, Interdisciplinary Center for Clinical Research, Friedrich-Alexander-University Erlangen-Nürnberg (FAU), Cauerstr. 3, 91058 Erlangen, Germany
[f]Department of Physics and Astronomy, and LaserLaB Amsterdam, Vrije Universiteit Amsterdam, De Boelelaan 1081, 1081 HV, Amsterdam, The Netherlands
[g]The Kavli Institute for Nanoscience Discovery, New Biochemistry Building, University of Oxford, South Parks Road, Oxford, OX1 3QU, UK

Content:

Supplementary Figures 1–7
References (for Supplementary Information only)

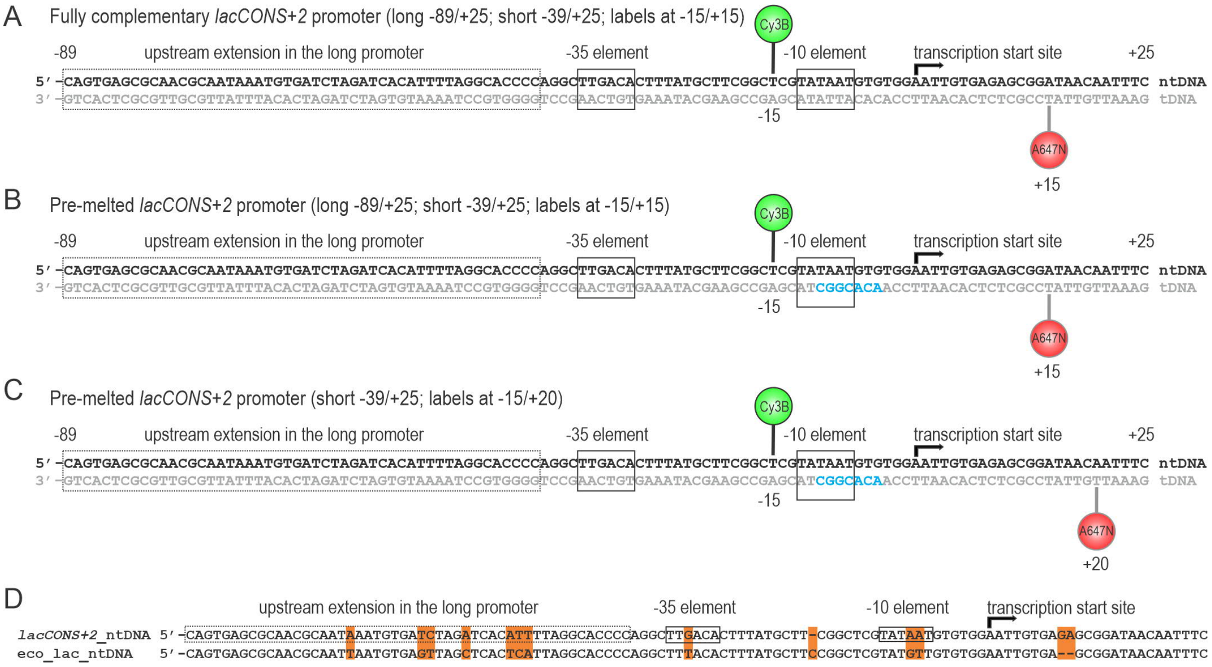

Figure S1. Promoter sequences used in the study. (A) The DNA sequences and fluorophore positions of the long and short fully double-stranded *lacCONS+2* promoters used in this study are shown. Non-template DNA (ntDNA) strand is in black, template DNA (tDNA) strand is in grey. Donor fluorophore Cy3B (green sphere) is attached to the -15 ntDNA thymine residue. Acceptor fluorophore ATTO647N (A647N, red sphere) is attached to the +15 tDNA thymine residue. (B) The long and short pre-melted versions of the *lacCONS+2* promoters were created by changing the template DNA sequence between the positions -10 and -4 as indicated in blue. (C) The short pre-melted version of the *lacCONS+2* promoter with acceptor ATTO647N label attached to the +20 tDNA thymine residue. This promoter was specifically used to demonstrate the transcriptional activity of formed RNAP–promoter complexes in Fig. S3. (D) Differences in the ntDNA sequence of the used *lacCONS+2* and native *E. coli lac* promoter are shown in orange. The substitutions in the CAP binding site in promoter upstream extension, the -35 element and the -10 element as well as the single basepair deletion in the -35/-10 spacer change these promoter elements to their consensus versions [53, 54].

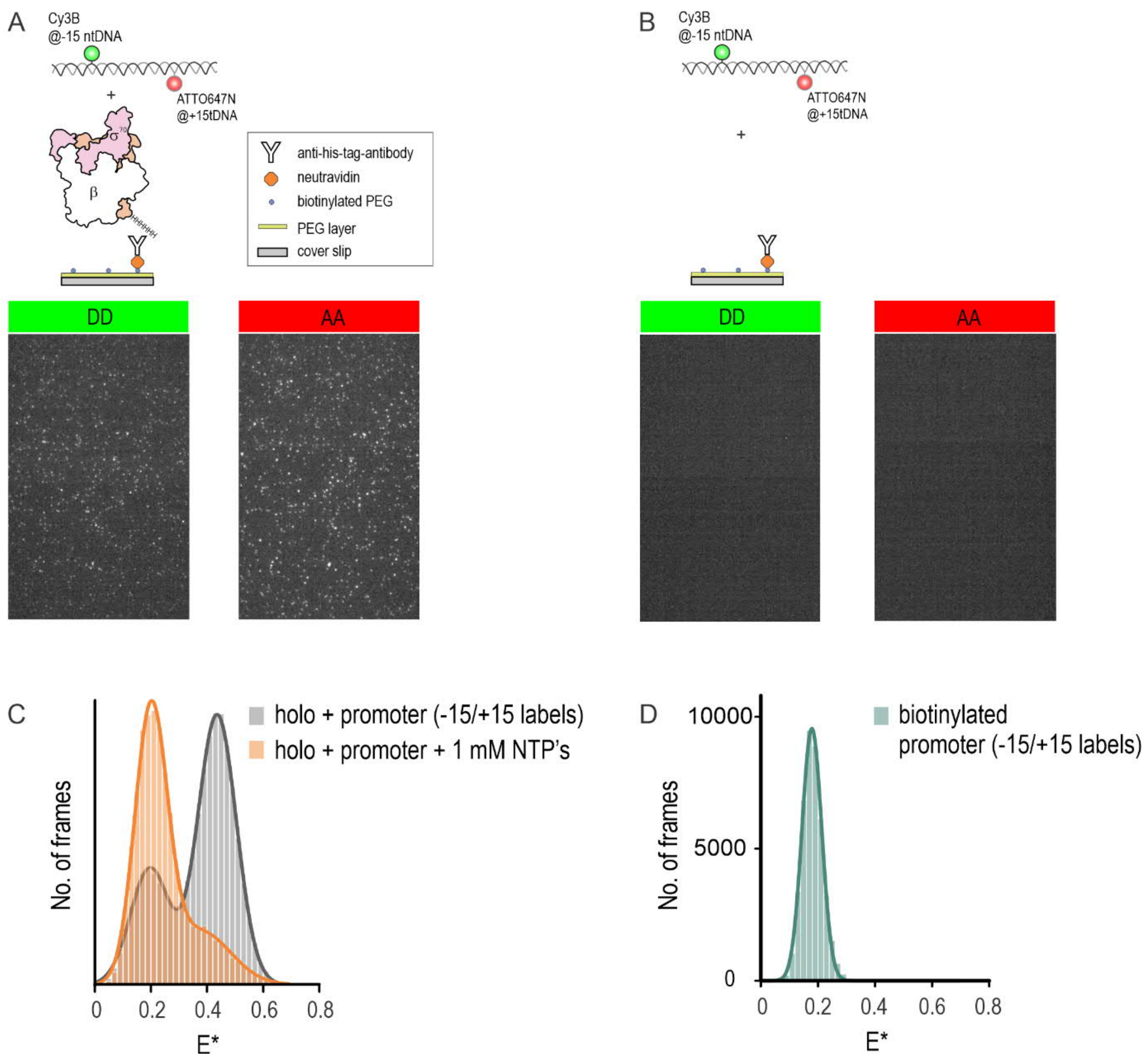

Figure S2. Specificity of promoter DNA binding to the surface-immobilised RNAP holoenzyme. (A) *E. coli* RNAP–σ^{70} holoenzyme was immobilised on the PEGylated coverslip. The immobilisation chemistry included PEG/biotin-PEG layer on the coverslip surface, neutravidin and biotinylated anti-His-tag-antibody. Short pre-melted *lacCONS+2* promoter (length -39/+25) labelled with Cy3B donor fluorophore at non-template DNA position -15 and ATTO647N acceptor fluorophore at template DNA position +15 was added to the reaction buffer and let to bind 5 min. The coverslip was then imaged with Nanoimager S microscope (ONI, Oxford, UK) using the total internal reflection mode. The objective magnification was 100X and numerical aperture 1.4. Cy3B fluorophore was imaged on the DD channel using 561 nm laser and ATTO647N fluorophore was imaged on the AA channel using 640 nm laser. The frame time per channel was 20 ms. The pixels of the raw images were 2 x 2 binned (sum) using Fiji software [55]; a single frame on each channel is shown. (B) Negative control experiment was performed as in panel A with the exception that the RNAP holoenzyme was omitted from the coverslip surface. (C) The E* histograms demonstrate the formation of RP_O (E~0.45) on the coverslip surface (grey, N=291 molecules). RP_O, i.e. the E~0.45 promoter state, disappears after 2 min incubation with 1 mM NTP's (orange, N=81) as the RNAP starts RNA synthesis and escapes the promoter that leads to promoter DNA conformation with low FRET efficiency. The E* values were extracted from each frame (20 ms) of the recorded movies. The promoter was long fully double-stranded lacCONS+2 with Cy3B at -15 ntDNA and ATTO647N at +15 tDNA. (D) The E* histogram of a biotinylated protein-free promoter (N=61). The data were collected as in panel C.

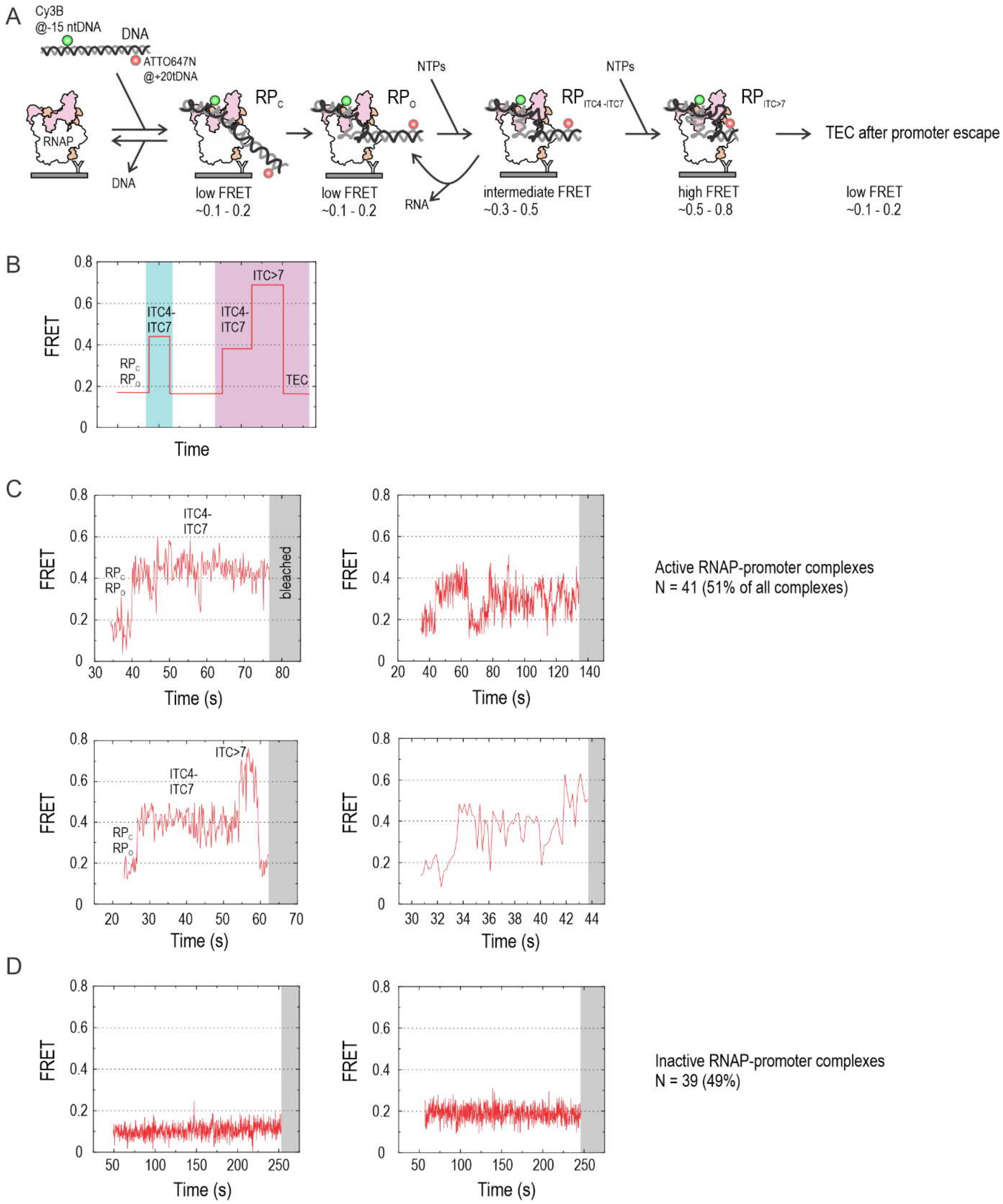

Figure S3. Transcription activity of surface-formed RNAP–promoter complexes. (A) The activity of the RNAP–promoter complexes was monitored using short pre-melted *lacCONS+2* promoter (length -39/+25) labelled with Cy3B fluorophore at non-template DNA position -15 and ATTO647N fluorophore at template DNA position +20. This previously developed labelling scheme creates a FRET ruler, which shows characteristic low FRET for the RP_O (and inactive RNAP–DNA complexes), intermediate FRET for initially transcribing complexes (ITCs) containing 4–7-mer RNA and high FRET for the ITCs containing >7-mer RNA, respectively. After RNAP escape from the promoter and the formation of transcription elongation complex (TEC) FRET returns to low level [56]. (B) Schematic FRET trajectory demonstrating the formation of RNAP–promoter complex at the beginning of FRET trace and subsequent increase in the FRET as the RP_O complex forms and commences RNA synthesis.

The FRET signatures highlighted with turquoise and purple demonstrate the events of abortive initiation and promoter escape, respectively. (C) Four experimental trajectories demonstrating transcription activity shortly after the formation of the RNAP–promoter complex. The RNAP was immobilised to the coverslip surface as described in Fig. S2. The imaging buffer contained 1 mM ATP, GTP, CTP and UTP; at the ~10 s time-point, 1 nM promoter DNA was added to the sample well. The frame time of the recordings was 200 ms with 100 ms ALEX excitation by green and red laser, respectively. Approximately 51% of all molecules (N=80 total molecules) showed FRET signatures indicating transcriptional activity. (D) Two experimental trajectories demonstrating the formation of inactive RNAP–promoter complexes. Approximately 49% of molecules showed FRET signatures of inactive RNAP–promoter complexes, i.e., static E* ~0.1–0.2.

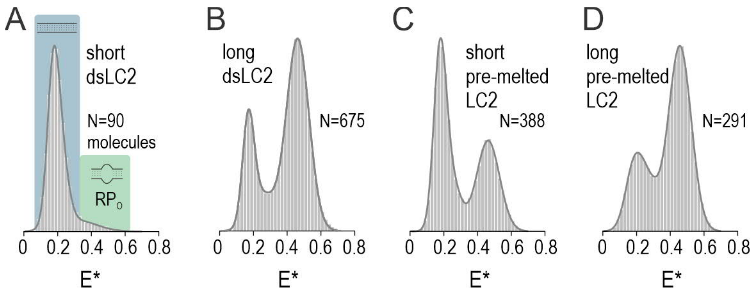

Figure S4. The formation efficiency of RP_O complex on different *lacCONS+2* promoters. The E* histograms reflect the formation efficiency of the open transcription bubble and RP_O complex (E*~0.45 peak) on (A) the short double-stranded LC2 promoter, (B) long dsLC2 promoter, (C) short pre-melted LC2 promoter and (D) long pre-melted LC2 promoter, respectively. RNAP–promoter complexes were recorded ~5 min after their initial formation on the coverslip surface. The FRET values were extracted from each frame (20 ms) of the recorded movies. The promoters were labelled with Cy3B at ntDNA position -15 and ATTO647N at tDNA position +15. Data statistics: short double-stranded LC2 promoter N=90 molecules and 35 400 frames; long double-stranded LC2 promoter N=675 molecules and 255 000 frames; short pre-melted LC2 promoter N=388 molecules and 99 600 frames; long pre-melted LC2 promoter N=291 molecules and 89 500 frames. Blue and green rectangles indicate expected E* values for the closed and open transcription bubble conformations, respectively. The E* ~0.2 species in the blue rectangle contain also unspecific RNAP–promoter complexes.

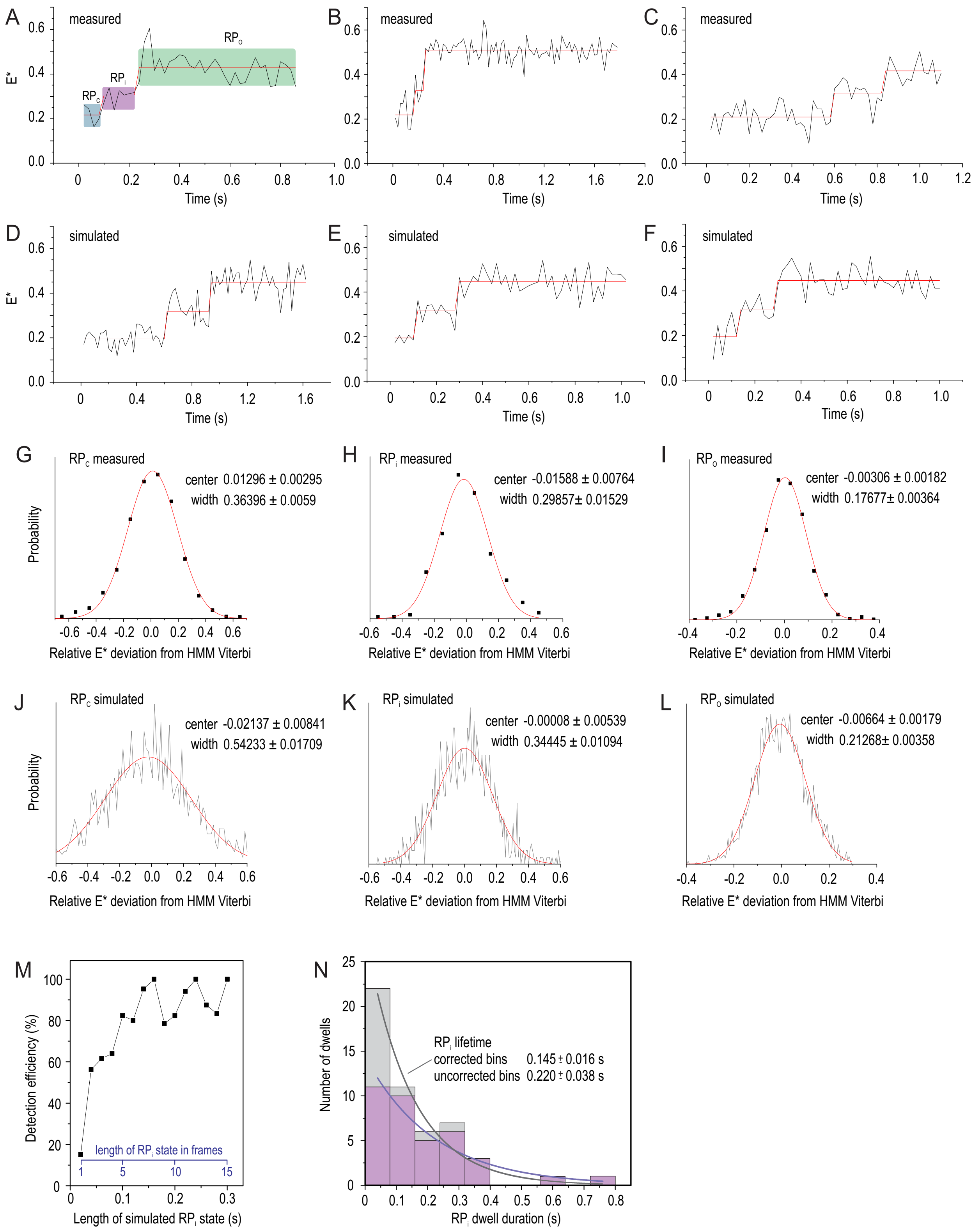

Figure S5. Detection efficiency of RP_i state. (A-C) Example trajectories showing the real RP_O formation events. The promoter binds to the holo at 0 s time-point forming RP_C, which isomerises via RP_i intermediate to RP_O state. Red line is the Viterbi from HMM fit to three state model. (D-F) Example simulated trajectories of RP_O formation events. (G) The relative deviation of measured E* values from Viterbi in each frame for the RP_C state in real data. Width parameter obtained from the fit to Gaussian equation is equal to 2 x noise. (H) The relative deviation of measured E* values from Viterbi in each frame for the RP_i state in real data. (I) The relative deviation of measured E* values from Viterbi in each frame for the RP_O state in real data. (J) The relative deviation of E* values from Viterbi in each frame for the RP_C state in simulated data. (K) The relative deviation of E* values from

Viterbi in each frame for the RP_i state in simulated data. (L) The relative deviation of E* values from Viterbi in each frame for the RP_O state in simulated data. (M) The detection efficiency of RP_i state in simulated trajectories is shown as a function of RP_i dwell length. (N) The mean lifetime of RP_i was obtained by fitting combined RP_i dwell time distribution of all promoters to mono-exponential equation. Purple and grey bins show data without or with correction for the missed RP_i events. To make the corrections for the missed events, the detection efficiency was set as 49% or 89% (based on data in panel M) for the shortest bin (0-0.08 s) of the experimental results and the other bins of the experimental results, respectively.

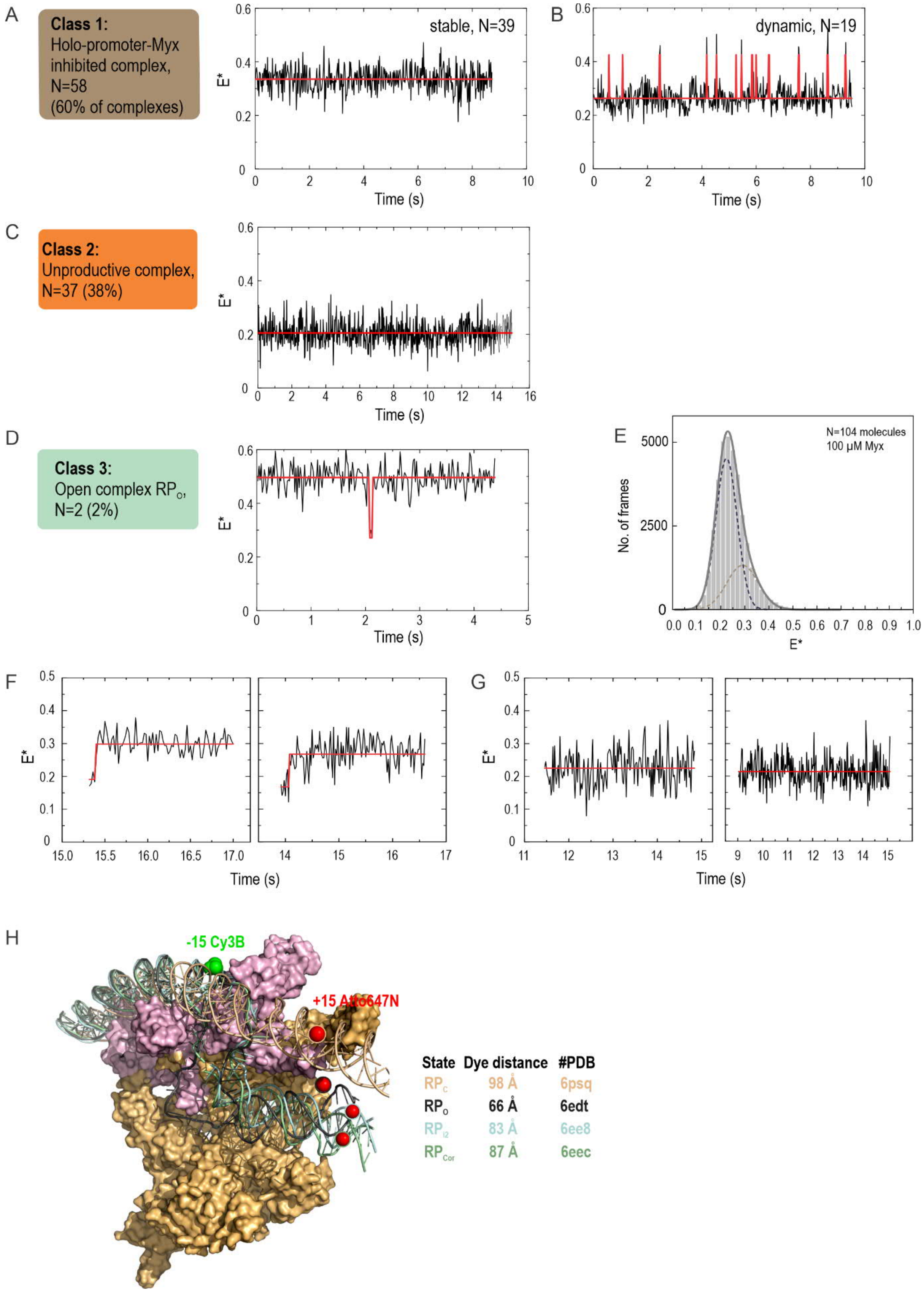

Figure S6. Characterisation of RNAP-promoter complexes formed in the presence of myxopyronin B inhibitor. (A) Preformed RNAP–promoter complexes were classified into three classes based on their FRET behavior. Class 1 molecules comprised of RNAP–promoter–Myx inhibition complexes, which

are characterised by E* ~0.3 state. (B) A subset of RNAP–promoter–Myx inhibited complexes sampled a short-lived higher E* state. Typically this higher E* state lasted only 1–2 frames (20–40 ms). (C) Class 2 molecules are characterised by stable E* ~0.2 state and probably constitute of non-productive RNAP-promoter complexes. (D) Class 3 molecules are RP_O complexes, which are identified based on their long-lived E* ~0.45 state. These few identified RP_O complexes probably formed after the dissociation of Myx inhibitor. The promoter in panels A–D was long dsLC2. (E) FRET efficiency histogram of the RNAP–promoter complexes preformed in the presence of Myx on long pmLC2 promoter. The fit of E* distribution to Equation 2 identified mean E* values of 0.224±0.002 and 0.290±0.045. (F) Two example trajectories demonstrating the real-time formation of initial RP_C complex (E*~0.2) and its subsequent isomerisation to E* ~0.3 state (N = 21). (G) Two example trajectories demonstrating the real-time formation of RNAP–promoter complex that remained in the E*~0.2 state for the entire duration of the trajectory (N = 45). The molecules in panels A–E were imaged ~5 min after the initial formation of the RNAP–promoter complexes on the coverslip surface. The molecules in panels F and G were imaged in real-time allowing the resolution of promoter binding and subsequent promoter conformation changes. The red solid line represents the Viterbi for a 2-state HMM. Myx concentration was 100 µM. (H) The location of Cy3B and ATTO647N fluorophores at the non-template DNA position -15 and template DNA +15, respectively, was modelled using FPS software [57] and indicated cryo-EM based RNAP structures. Noteworthy, corallopyronin was suggested to trap RNAP to a state with close similarity to a normal intermediate RP_{i2} in the RP_O formation pathway [58]. The cryo-EM structure of RP_C was obtained using *E. coli* proteins [59] whereas RP_{Cor}, RP_{i2} and RP_O structures were obtained using *M. tuberculosis* proteins [58]. The distance between the average positions of modelled -15/+15 fluorophores in each RNAP state is shown. The protein elements, i.e., β' RNAP subunit (light orange) and σ factor (pink), are shown as in the RP_O cryo-EM model [58].

A

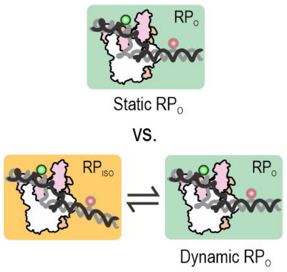

B

	stable RP$_O$	dynamic RP$_O$
short dsLC2	76.8% (*N*=334 molecules)	23.2% (101)
long dsLC2	85.3% (297)	14.7% (51)

molecules pooled from three independent experiments

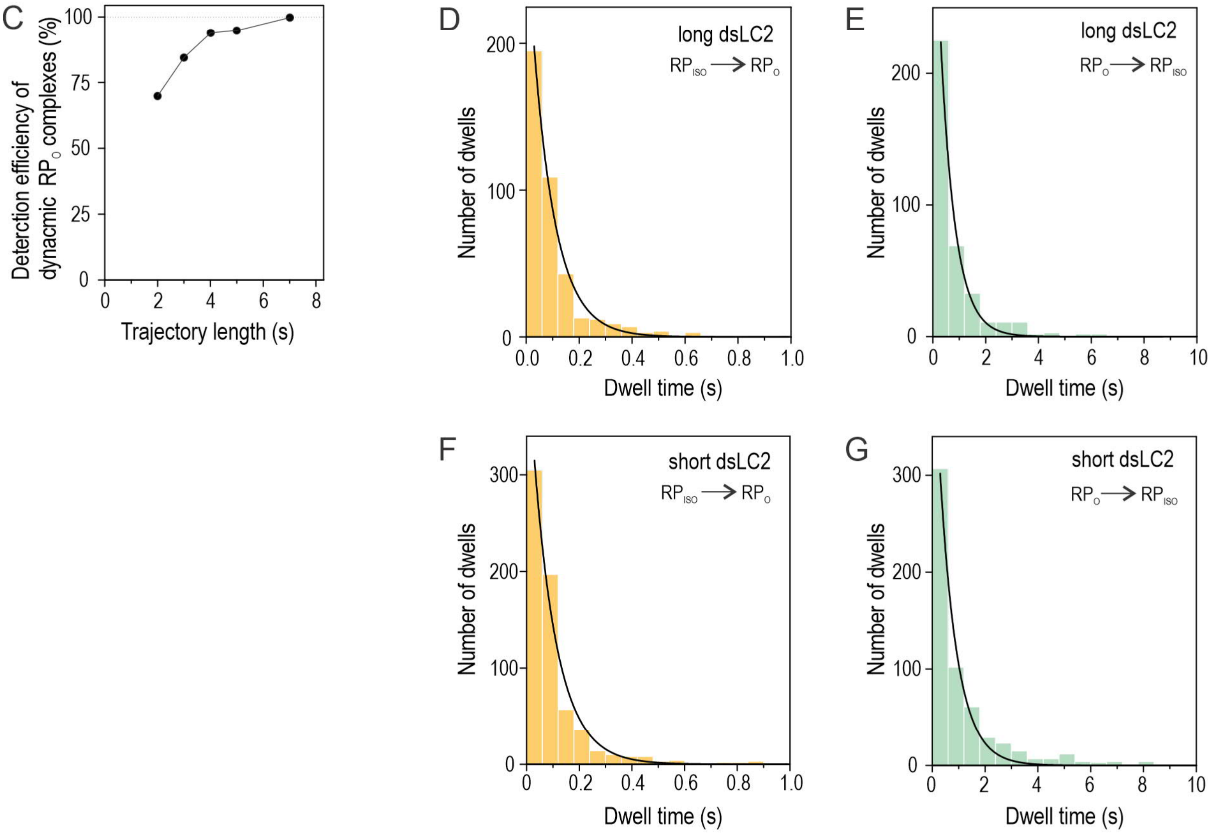

H

	RP$_{ISO}$ → RP$_O$	τ_{RPiso} (s)	RP$_O$ → RP$_{ISO}$	τ_{RPo} (s)
short dsLC2		0.090±0.006		0.663±0.041
long dsLC2		0.085±0.004		0.559±0.027

Figure S7. Effect of αCTD–promoter interactions on the reaction pathway branching and the rates of transcription bubble dynamics. (A) Dynamic RP$_O$ complex samples RP$_{ISO}$ state while static RP$_O$ does not show any FRET transitions. (B) The formation frequency of stable and dynamic RP$_O$ complexes on short (-39/+25) and long (-89/+25) *lacCONS+2* promoters. (C) The detection efficiency of dynamics RP$_O$ complexes was estimated using simulated trajectories with different lengths. 92% of experimental trajectories have length ≤3 s (D–G) The dwell time distribution of RP$_{ISO}$ (yellow) and RP$_O$ (green) states in the population of dynamic RP$_O$. The dwell time distributions were fit to mono-exponential equation. (H) The mean lifetime (*t*) of the RP$_{ISO}$ (yellow) or RP$_O$ (green) state. RP$_O$ complexes were prepared in solution at elevated temperature (37°C), challenged with competitor (heparin) and immobilised on the coverslip surface for smFRET analysis at 22°C.

References (for Supplementary Information only)

53. Ebright,R.H., Yon W. & Ebright,A.G. (1989) Consensus DNA site for the *Escherichia coli* catabolite gene activator protein (CAP): CAP exhibits a 450-fold higher affinity for the consensus DNA site than for the *E.coli* lac DNA site. *Nucleic Acids Res.*, 17, 10295–10305.

54. Mekler,V., Kortkhonjia,E., Mukhopadhyay,J., Knight,J., Revyakin,A., Kapanidis,A.N., Niu,W., Ebright,Y.W., Levy,R. & Ebright,R.H. (2002) Structural organization of bacterial RNA polymerase holoenzyme and the RNA polymerase-promoter open complex. *Cell*, 108, 599–614.

55. Schindelin,J., Arganda-Carreras,I., Frise,E., Kaynig,V., Longair,M., Pietzsch,T., Preibisch,S., Rueden,C., Saalfeld,S., Schmid,B., Tinevez,J.-Y., White,D.J., Hartenstein,V., Eliceiri,K., Tomancak,P. & Cardona,A. (2012) Fiji: An open-source platform for biological-image analysis. *Nat. Methods*, 9, 676–682.

56. Duchi,D., Bauer,D.L.V., Fernandez,L., Evans,G., Robb,N., Hwang,L.C., Gryte,K., Tomescu,A., Zawadzki,P., Morichaud,Z., Brodolin,K. & Kapanidis,A.N. (2016) RNA Polymerase Pausing during Initial Transcription. *Mol. Cell*, 63, 939–950.

57. Kalinin,S., Peulen,T., Sindbert,S., Rothwell,P.J., Berger,S., Restle,T., Goody,R.S., Gohlke,H. & Seidel,C.A.M. (2012) A toolkit and benchmark study for FRET-restrained high-precision structural modeling. *Nat. Methods*, 9, 1218–1225.

58. Boyaci,H., Chen,J., Jansen,R., Darst,S.A. & Campbell,E.A. (2019) Structures of an RNA polymerase promoter melting intermediate elucidate DNA unwinding. *Nature*, 565, 382–385.

59. Chen,J., Chiu,C., Gopalkrishnan,S., Chen,A.Y., Olinares,P.D.B., Saecker,R.M., Winkelman,J.T., Maloney,M.F., Chait,B.T., Ross,W., Gourse,R.L., Campbell,E.A. & Darst,S.A. (2020) Stepwise Promoter Melting by Bacterial RNA Polymerase. *Mol. Cell*, 78, 275-288.e6.
